# The “where” of social attention: Head and body direction aftereffects arise from representations specific to cue type and not direction alone

**DOI:** 10.1080/17588928.2015.1049993

**Published:** 2015-06-16

**Authors:** Rebecca P. Lawson, Andrew J. Calder

**Affiliations:** 1 Wellcome Trust Centre for Neuroimaging, Insitiute of Neurology, University College London, London, UK; 2 Medical Research Council Cognition and Brain Sciences Unit, Cambridge, UK

**Keywords:** Social attention cues, Adaptation, Head and body direction

## Abstract

Human beings have remarkable social attention skills. From the initial processing of cues, such as eye gaze, head direction, and body orientation, we perceive where other people are attending, allowing us to draw inferences about the intentions, desires, and dispositions of others. But before we can infer *why* someone is attending to something in the world we must first accurately represent *where* they are attending. Here we investigate the “where” of social attention perception, and employ adaptation paradigms to ascertain how head and body orientation are visually represented in the human brain. Across two experiments we show that the representation of two cues to social attention (head and body orientation) exists at the category-specific level. This suggests that aftereffects do not arise from “social attention cells” discovered in macaques or from abstract representations of “leftness” or “rightness.”

Single-cell recording in non-human primates and, more recently, adaptation research in humans, is beginning to reveal how gaze, head, and body cues to social attention are visually represented in the human brain. Electrophysiological recording in the anterior superior temporal sulcus (STS) of macaques has demonstrated separate cell populations responsive to different gaze directions, head directions and body orientations. It was proposed that these direction-selective cells may play a role in analyzing the direction of other people’s attention signaled by these cues (Harries & Perrett, 1991; Perrett, Hietanen, Oram, Benson, & Rolls, 1992; Perrett et al., 1991; Wachsmuth, Oram, & Perrett, 1994). Recent behavioral adaptation research has demonstrated that, akin to monkeys, humans have functionally distinct (and hence adaptable) mechanisms coding left and right gaze directions (Calder, Jenkins, Cassel, & Clifford, 2008; Jenkins, Beaver, & Calder, 2006). After adapting to a single gaze direction (e.g., left) participants showed striking repulsive aftereffects, with the perception of subsequently seen leftward gaze shifted toward “direct.” Similar adaptation aftereffects have also been found for directionally oriented heads (Fang & He, 2005; Lawson, Clifford, & Calder, 2011) and bodies (Lawson, Clifford, & Calder, 2009; Taylor, Wiggett, & Downing, 2010).

Adaptation studies are readily interpreted as evidence for the existence of directionally-selective cells tuned to different types of social cue (e.g., eyes, heads and bodies). However, a proportion of the cells recorded from in the macaque STS responded to more than one type of cue (e.g., similar responses for heads and bodies when presented in isolation) oriented in the same direction (e.g., facing left) (Perrett et al., 1992). These cells were referred to as “social attention cells” because they were selective to one particular direction irrespective of the type of social cue signaling that direction. Perrett and colleagues posit that these cells may encode the locus of another’s attention by combining the outputs of cells which analyze the direction of eye gaze, head direction, and body orientation separately, responding an equivalent amount to each cue presented in isolation. However, when multiple directional cues are present in the same stimulus a hierarchy of inhibitory connections modulate the response of the social attention cells, whereby eye gaze cues will override head direction cues but not vice versa, and head cues in turn override body orientation cues but not vice versa (Perrett et al., 1992). This model therefore not only allows attention direction to be computed when multiple cues are visible, but also under a variety of different viewing conditions.

It is unknown whether the behavioral adaptation aftereffects for head and body direction (Lawson et al., 2009, 2011) arise from analogous “social attention cells” that respond equally to both heads *and* bodies oriented in the same direction, or from separate classes of direction-selective cells, lower in the processing stream, responsive to heads *or* bodies alone. If the former were the case then the within-category adaptation—heads adapting heads and bodies adapting bodies—observed in previous research should extend between social categories to an equivalent extent (i.e., bodies presented in isolation should adapt heads presented in isolation and vice versa). Alternatively, if head and body adaptation aftereffects occur at the level of cells coding head direction and body orientation alone, then there should be no transfer of adaptation between these categories. Here we address, for the first time, the level at which head and body direction aftereffects operate.

In Experiment 1, categorization of head direction is measured following adaptation to alternating 20º left and 20º right oriented adaptors. There were three adaptor conditions: Same-social adaptor (heads), different-social adaptor (bodies), and a control non-social adaptor condition (chairs). In Experiment 2, body direction categorization is measured following adaptation to the same three adaptor conditions, only in the context of body orientation categorization; bodies were now the same-social adaptor and heads were the different-social adaptor. Alternating left/right adaptation has previously been shown to produce effects comparable in magnitude to simple leftward (or rightward) adaptation, but with a single adaptation condition rather than two (Calder et al., 2008). As we had no specific interest in direction selectivity, and in consideration of time, we opted for alternating left/right adaptation in both experiments. For both experiments it was predicted that, consistent with previous research (Lawson et al., 2009, 2011), participants would show an increased tendency to call small angles of left and right oriented stimuli “direct” following adaptation in the same-social adaptor condition. Additionally, if these aftereffects are occurring at the level of “social attention cells,” then adaptation to the different-social adaptor should also produce comparable aftereffects. Finally, if these adaptation aftereffects are occurring at the level of non-category-specific object representations coding any object’s orientation, then the non-social adaptor condition should also produce measurable aftereffects in the same predicted direction.

## EXPERIMENT 1—HEAD DIRECTION

### Methods

#### Participants

Fifteen right-handed volunteers from the MRC Cognition and Brain Sciences Unit (CBU) volunteer panel (six female; mean age 26.3 years, *SD* = 1.7) participated in return for payment. All had normal or corrected vision. This study was approved by the Cambridge Psychology Research Ethics Committee (CPREC).

#### Materials

**Figure 1.**
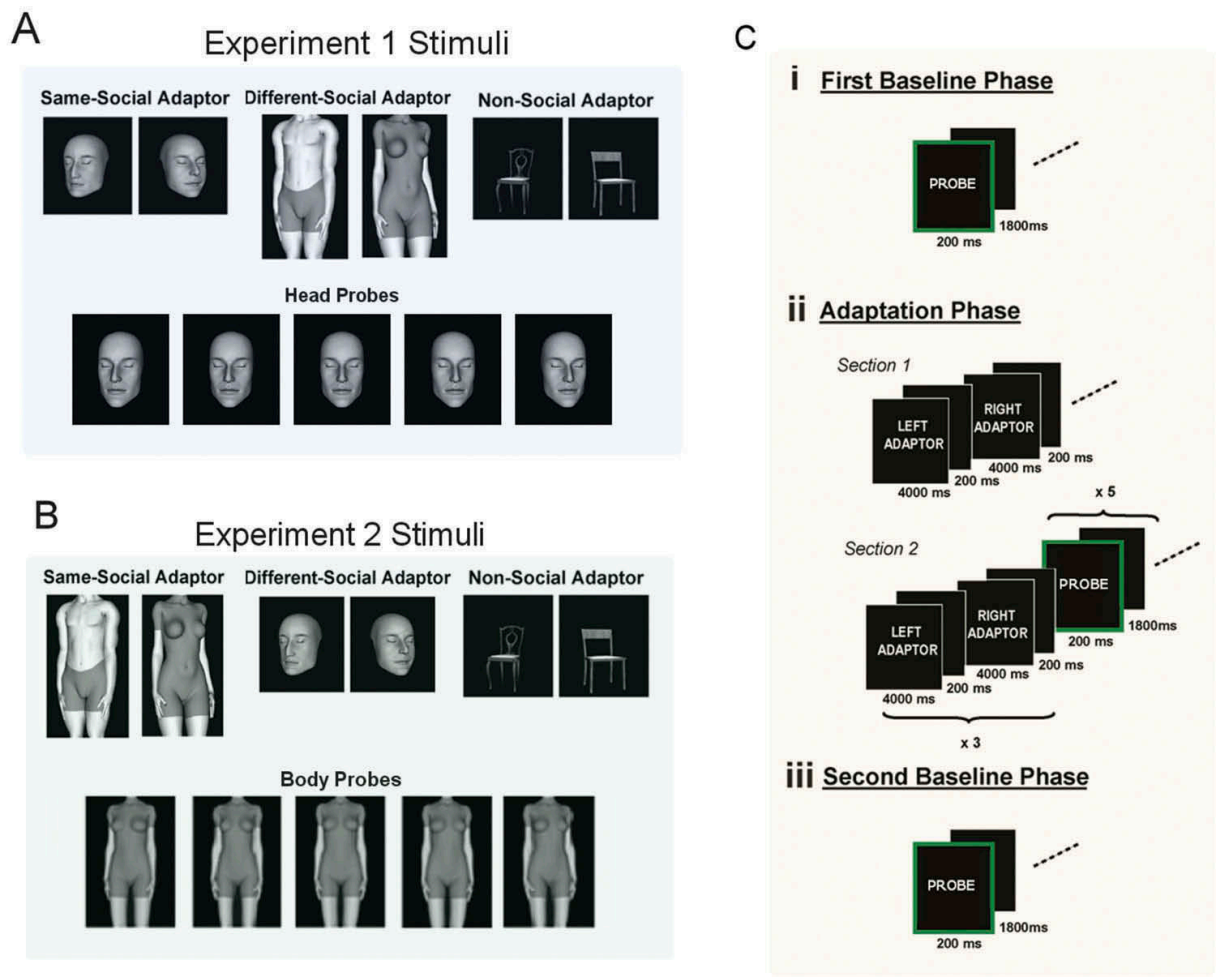
Sample stimuli, trial format, and procedure for both experiments. (A) From experiment 1—Examples of the probe head images and adaptation stimuli for each of the three adaptation conditions and (B) from experiment 2—Examples of probe body images the adaptation stimuli for each of the three adaptation conditions. Images depict only a small number of identities and are not to scale, for illustration purposes only. (C) Both experiments had a three-phase format comprising (i) a pre-adaptation baseline phase (baselines 1 and 2) (ii) an adaptation phase, and (iii) a post-adaptation baseline phase (baselines 3 and 4) identical to the first. In the baseline phases participants categorized the direction (left, direct, or right) of probe images (heads or bodies; green border) oriented in one of five directions. The adaptation phase consisted of two sections. In section 1 participants adapted to an alternating series of 20º left and 20º right oriented adaptors (either, heads, bodies, or chairs) and in section 2 the baseline phase was repeated with every five probe heads preceded by six top-up adaptor images. Participants completed these three phases three times, once for each adaptation condition (heads, bodies, and chairs) in a counterbalanced order for both experiments. See the text for full details of the procedures.

The experimental stimuli were gray-scale, computer-simulated images of human heads, bodies, and chairs created using DAZ 3D software (version 3.0.1.1). The probe images depicted six head identities with their eyes closed (three male and three female), each facing in five directions: 8° left, 4° left, 0° direct, 4° right, and 8° right. The adaptation stimuli consisted of six different head identities with their eyes closed (same-social adaptor condition), six body identities (different-social adaptor condition), or six individual examples of chairs (non-social adaptor condition); each oriented 20° to the left and 20° to the right (Figure 1a). Probe stimuli measured 3 cm vertically and 2.5 cm horizontally, subtending a visual angle of approximately 3° X 2.5° at a viewing distance of 57 cm. Adaptation stimuli were 25% bigger to disrupt low-level visual features between the adaptor and probes in the same-social condition. A head rest was used throughout to ensure a constant viewing distance and head position.

#### Design and procedure

For each adaptation condition (heads, bodies, and chairs) the experiment comprised three phases (Figure 1c): A pre-adaptation baseline phase (baselines 1 and 2), an adaptation phase which comprised two sections, and a second post-adaptation baseline phase (baselines 3 and 4). There were three adaptation conditions: Same-social (heads), different-social (bodies), and non-social (chairs). Participants completed each of the three conditions in a counterbalanced order, in a single experimental session, with separate pre- and post-adaptation baselines before and after each adaptation condition. Testing sessions took approximately one hour with breaks as requested.

#### First baseline phase

This comprised two identical blocks (baselines 1 and 2) each showing six head identities twice across five orientations (8° left, 4° left, 0° direct, 4° right, and 8° right; 60 stimuli in total). Each trial consisted of a probe head for 200 ms, and then a 1800-ms Interstimulus Interval (ISI). Participants categorized the head direction as “left,” “direct,” or “right.” Presentation order was randomized. Baseline 1 was used to familiarize participants with the task and was disregarded as practice.

#### Adaptation phase

The adaptation phase comprised two sections.

*Section 1* comprised a series of adaptor images presented for 4000 ms each. Depending on the condition, adaptor images were either heads, bodies, or chairs oriented 20° to the left and 20° to the right (38 images in total). Adjacent adaptors never showed the same identity and a 200-ms ISI served to eliminate any “apparent” motion. Participants performed a dot detection task, which occurred on 8% of trials, to ensure attention throughout.

*Section 2* contained the same probe heads as the baseline blocks with exactly the same presentation times (200 ms each with a 1800-ms ISI for response logging). Again, participants categorized the direction as “left,” “direct,” or “right.” However, preceding every five probe images were six alternating left/right “top-up adaptors” to maintain adaptation. Top-up images were presented for 1000 ms each, followed by a 200-ms ISI. Top-ups were always a different size to the following probe stimuli (and a different identity in the same-social condition) and identities were counterbalanced across trials.

#### Second baseline phase

This was identical to the first baseline phase, comprising two baselines (3 and 4). Baseline 3 served to dissipate any remaining effects of adaptation which have been shown to persist for up to 385 seconds following eye gaze adaptation (Kloth & Schweinberger, 2008) and was not included in the analysis.

### Results

As per convention (Calder et al., 2007, 2008; Jenkins et al., 2006; Lawson et al., 2009, 2011; Schweinberger, Kloth, & Jenkins, 2007) data are summarized as mean percentage of “direct” responses to the probe heads and adaptation is measured as a change in “direct” responses between adaptation and baseline phases. Greenhouse-Geisser correction was used when appropriate. All the analyses of variance (ANOVAs) reported, and *t*-test comparisons were Bonferroni corrected (*p* < .01, corrected for five comparisons) with uncorrected *p* values reported throughout. Prior to analysis, data were arcsine-transformed to stabilize variance of the proportion measures, which showed a range of values including values close to ceiling and floor. The patterns of results were identical to those found using non-transformed data.

**Figure 2.**
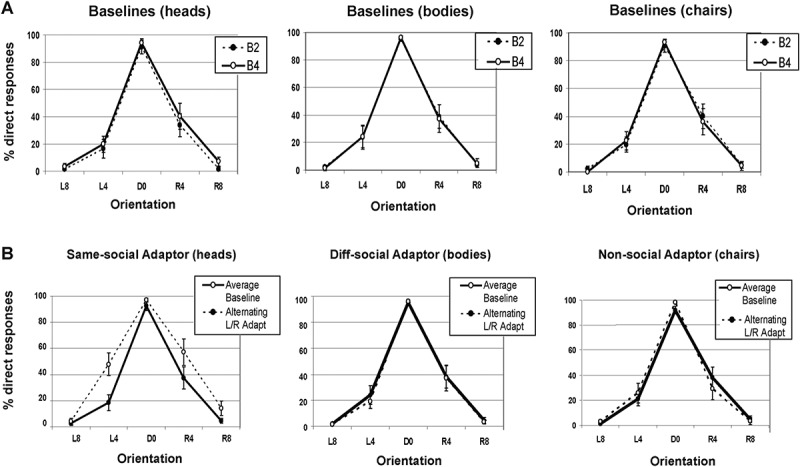
Results of experiment 1. Separate graphs show the mean percentage of “direct” responses to different head direction probes for (A: Top row) baselines 2 and 4 in the context of adaptation to heads, bodies, and chairs and (B: Bottom row) following adaptation to an alternating sequence of same-social adaptors (heads), different-social adaptors (bodies), and non-social adaptors (chairs) oriented 20° to the left and 20° to the right (overall average performance on baselines 2 and 4 across all three conditions is also shown). Error bars represent standard errors. Head orientations are labeled as: L8 = Left 8°, L4 = Left 4°, D0 = Direct 0°, R4 = Right 4°, and R8 = Right 8°. B2 = Baseline 2 and B4 = Baseline 4.

Baselines 2 and 4 (Figure 2a) were submitted to a 3x2x5 ANOVA investigating the factors of adaptation condition (heads, bodies, and chairs), baseline (2 and 4) and orientation (8° Left, 4° Left, 0° Direct, 4° Right, and 8° Right). This analysis found no main effect of condition or baseline (*F*s < 1), but, as expected, found a significant main effect of head orientation (*F*(2.28, 31.90) = 121.88, *MSE* = 1272.81, *p* < .001), reflecting more accurate categorization of direct and 8° head directions than 4° head directions. There was no condition x baseline interaction (*F* = 2.86, *p* > .07) or any other interactions between these factors (all *F*s < 1) thus demonstrating that baseline performance did not differ as a function of which adaptation condition the baselines corresponded to or between baselines 2 and 4. Consequently, the remaining analyses compare the effects of each adaptation condition (heads, bodies, and chairs) against the average of their individual corresponding baselines (2 and 4). All subsequent references to “average baselines” refer to the average baseline corresponding to each adaptation condition.

Following adaptation to left and right oriented heads (same-social adaptor), but not adaptation to left and right oriented bodies (different-social adaptor) or chairs (non-social adaptor), participants showed an increased tendency to categorize 4º left and 4º right oriented probe heads as “direct” (Figure 2b). A 3x2x5 repeated measures ANOVA with factors of adaptor type (heads, bodies, chairs), adaptation (alternating left/right, average baseline), and orientation (8° Left, 4° Left, 0° Direct, 4° Right, and 8° Right) showed a significant main effect of adaptor type (*F*(2, 28) = 8.67, *MSE* = 156.84, *p *< .001), adaptation (*F*(1, 14) = 5.22, *MSE* = 214.82, *p *< .04) and orientation (*F*(2.25, 31.45) = 128.52, *MSE* = 1224.29, *p *< .001). There was a significant adaptor type x orientation interaction (*F*(4.22, 59.09) = 2.93, *MSE* = 133.16, *p *< .05), but no adaptation x orientation interaction (*F* = 1.86, *p* < .13). Crucially, this analysis showed a significant adaptor type x adaptation interaction (*F*(1.33, 18.56) = 18.10, *MSE* = 117.57, *p *< .001) and a significant three-way interaction between adaptor type, adaptation, and orientation (*F*(4.49, 62.84) = 5.49, *MSE* = 116.02, *p *< .001), suggesting that the effects of adaptation were significantly different for the three adaptor types.

To investigate these interactions further, individual 2x5 ANOVAs investigating adaptation (alternating left/right, average baseline) and head orientation were conducted for each of the adaptation conditions. As expected, all three adaptor types produced a significant main effect of orientation (Heads *F*(4, 56) = 96.26, *MSE* = 297.02, *p* < .001; Bodies *F*(2.12, 29.93) = 115.76, *MSE* = 256.56, *p* < .001, and Chairs *F*(2.62, 36.70) = 111.07, *MSE* = 418.88, *p* < .001). Consistent with previous research (Lawson et al., 2011), head adaptors produced a significant main effect of adaptation (*F*(1, 14) = 26.57, *MSE* = 146.30, *p* < .001) whereas bodies and chairs did not (*F*s < 1). Head adaptors also produced a significant adaptation x orientation interaction (*F*(4, 56) = 9.02, *MSE* = 53.59, *p *< .001), as did chairs (*F*(4, 56) = 5.55, *MSE* = 55.43, *p *< .002) but bodies did not (*F* < 1). Paired *t*-tests investigating the interaction between adaptation and head direction for both head adaptors and chair adaptors showed that, consistent with the predictions of this paper, adaptation to the same-social adaptor (heads) produced an *increased* tendency to call 4º left and 4º right probe heads “direct,” L4° *t*(14) = 5.03, *p* < .001; R4° *t*(14) = 4.55, *p* < .001. There was a borderline increase in “direct” responses to 8º right probes that did not survive Bonferroni correction, R8º *t*(14) = 2.51, *p* = .02, and no significant effect for direct, *t*(14) = 1.76 or left 8º probes *t*(14) = 1.09, *p*s* *> .10. However, the effects for the chair adaptors did not follow the predictions of adaptation (i.e., increased “direct” responses to small angles of left and right facing probes) therefore this effect is perhaps unlikely to reflect adaptation per se (see Discussion). The non-social adaptor (chairs) produced an increase in “direct” responses to direct facing heads, *t*(14) = 3.09, *p* < .01 and a borderline but non-significant *decrease* in “direct” responses to 4º right facing heads, *t*(14) = −2.52, *p* > .2. There were no significant differences for any other orientation, L8º *t*(14) = 1.03, L4º *t*(14) = 0.85, R8º *t*(14) = 0.96, *p*s > .32).

Thus far, this analysis shows that the same-social adaptor (heads) produces the predicted increased tendency to call 4º left and 4º right probes “direct,” whereas body and chair adaptors do not. However, to ensure that the *effects of adaptation* produced by the head adaptors is significantly different from the body adaptors and the chair adaptors, three additional repeated measures ANOVAs were conducted to compare the effects of adapting to chairs with bodies, heads with bodies, and finally, heads with chairs. The results can be seen in Table 1 and confirm that only heads compared to either of the other adaptation conditions produces a significant three-way adaptor type x adaptation x orientation interaction. When comparing the effects of adapting to bodies with adapting to chairs, there is no adaptor type x adaptation interaction or three-way adaptor type x adaptation x orientation interaction, thus the effects of non-social (chairs) and different-social (bodies) adaptors are indistinguishable.

**TABLE 1:** Results of a three additional repeated measures ANOVAs from experiment 1 and experiment 2. ** = *p* < .005, * = *p* < .05, ^1^ = trend.

Experiment 1						Experiment 2					
ANOVA	Adaptor type (heads, bodies) x adaptation x orientation					ANOVA	Adaptor type (bodies, chairs) x adaptation x orientation				
Main Effects	F	d.f	d.f. error	MSE	p-value	Main Effects	F	d.f	d.f. error	MSE	p-value
*Adaptor Type*	13.24	1.00	14.00	147.15	.003**	*Adaptor Type*	1.39	1.00	14.00	157.30	.259
*Adaptation*	9.09	1.00	14.00	170.70	.009*	*Adaptation*	17.54	1.00	14.00	112.36	.001**
*Orientation*	119.39	2.24	31.41	866.77	.001*	*Orientation*	288.45	2.64	36.89	257.64	.001**
Interactions						Interactions					
Adaptor Type * Adaptation	22.99	1.00	14.00	103.54	.001**	Adaptor Type * Adaptation	5.64	1.00	14.00	254.01	.032*
Adaptor Type * Orientation	3.65	4.00	56.00	67.40	.01*	Adaptor Type * Orientation	1.39	4.00	56.00	74.90	.249
Adaptation * Orientation	2.24	4.00	56.00	68.10	.076	Adaptation * Orientation	3.57	4.00	56.00	86.22	.012*
Adaptor Type * Adaptation * Orientation	5.25	4.00	56.00	71.58	.001**	Adaptor Type * Adaptation * Orientation	2.87	4.00	56.00	63.58	.031*
*ANOVA*	*Adaptor type (heads, chairs) x adaptation x orientation*					*ANOVA*	*Adaptor type (bodies, heads) x adaptation x orientation*				
*Main Effects*	*F*	*d.f*	*d.f. error*	*MSE*	*p-value*	*Main Effects*	*F*	*d.f*	*d.f. error*	*MSE*	*p-value*
*Adaptor Type*	21.01	1.00	14.00	101.18	.001**	*Adaptor Type*	2.63	1.00	14.00	141.99	.127
*Adaptation*	9.49	1.00	14.00	222.36	.008*	*Adaptation*	24.36	1.00	14.00	119.47	.001**
*Orientation*	115.78	2.50	35.06	810.62	.001**	*Orientation*	214.95	2.65	37.04	357.08	.001**
Interactions						Interactions					
Adaptor Type * Adaptation	80.26	1.00	14.00	22.45	.001**	Adaptor Type * Adaptation	3.21	1.00	14.00	249.64	.095
Adaptor Type * Orientation	5.09	4.00	56.00	64.06	.001**	Adaptor Type * Orientation	12.29	4.00	56.00	87.81	.023*
Adaptation * Orientation	3.77	4.00	56.00	66.10	.009*	Adaptation * Orientation	16.70	4.00	56.00	78.83	.005**
Adaptor Type * Adaptation * orientation	12.63	4.00	56.00	42.93	.001**	Adaptor Type * Adaptation * orientation	2.44	4.00	56.00	81.97	.*058^1^*
*ANOVA*	*Adaptor type (bodies, chairs) x adaptation x orientation*					*ANOVA*	*Adaptor type (heads, chairs) x adaptation x orientation*				
*Main Effects*	*F*	*d.f*	*d.f. error*	*MSE*	*p-value*	*Main Effects*	*F*	*d.f*	*d.f. error*	*MSE*	*p-value*
*Adaptor Type*	0.02	1.00	14.00	222.20	.900	*Adaptor Type*	0.22	1.00	14.00	93.10	.645
*Adaptation*	0.08	1.00	14.00	116.54	.780	*Adaptation*	2.07	1.00	14.00	125.09	.172
*Orientation*	133.13	2.20	30.76	822.57	.001**	*Orientation*	239.83	2.13	29.79	420.10	.001**
Interactions						Interactions					
Adaptor Type * Adaptation	0.37	1.00	14.00	107.89	.550	Adaptor Type * Adaptation	1.56	1.00	14.00	58.40	.232
Adaptor Type * Orientation	0.56	4.00	56.00	79.30	.690	Adaptor Type * Orientation	1.13	4.00	56.00	68.91	.350
Adaptation * Orientation	3.26	4.00	56.00	95.67	.04*	Adaptation * Orientation	3.15	4.00	56.00	80.12	.021*
Adaptor Type * Adaptation * Orientation	1.92	4.00	56.00	80.79	.120	Adaptor Type * Adaptation * Orientation	0.32	4.00	56.00	65.47	.862

## EXPERIMENT 2—BODY ORIENTATION

### Methods

#### Participants

Fifteen different right-handed volunteers from the MRC Cognition and Brain Sciences Unit (CBU) volunteer panel (six female; mean age 26.1 years, *SD* = 3.6) participated in return for payment. All had normal or corrected vision.

#### Materials

The experimental stimuli were created using the same software as Experiment 1. The probe images depicted six body identities (three male and three female; Figure 1b), each facing in five directions: 12° left, 6° left, 0° direct, 6° right, and 12° right, consistent with the body orientations used in previous research (Lawson et al., 2009). Heads and legs, from the knee down, were removed using Adobe Photoshop software (version 7.0.1) so that neither could provide a cue to the body direction. The adaptation stimuli are identical to Experiment 1 (Figure 1b). The image sizes and visual angles of the adaptation and probe stimuli were identical to Experiment 1.

#### Design and procedure

The design and procedure was identical to Experiment 1 (Figure 1c).

## RESULTS

**Figure 3.**
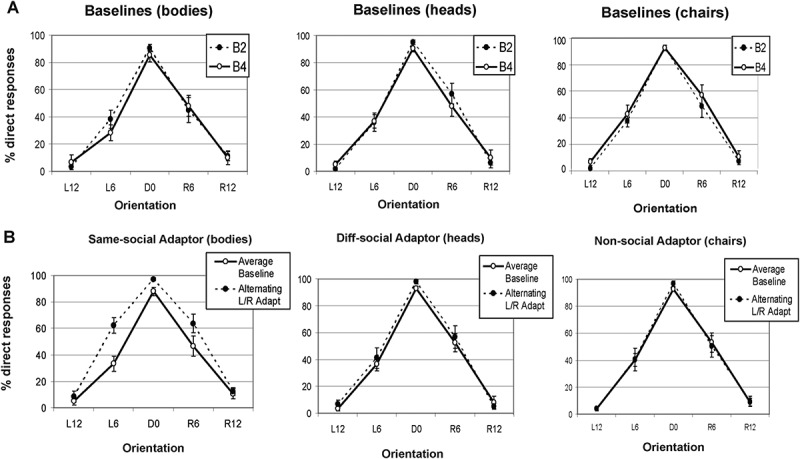
Results of experiment 2. Separate graphs show the mean percentage of “direct” responses to different body direction probes for (A: Top row) baselines 2 and 4 in the context of adaptation to heads, bodies, and chairs and (B: Bottom row) following adaptation to an alternating sequence of same-social adaptors (bodies), different-social adaptors (heads), and non-social adaptors (chairs) oriented 20° to the left and 20° to the right (overall average performance on baselines 2 and 4 across all three conditions is also shown). Error bars represent standard errors. Body orientations are labeled as: L12 = Left 12°, L6 = Left 6°, D0 = Direct 0°, R6 = Right 6° and R12 = Right 12°. B2 = Baseline 2 and B4 = Baseline 4.

A 3x2x5 ANOVA investigating the factors of adaptation condition, baseline, and orientation determined that baseline performance did not differ as a function of which adaptation condition the baselines corresponded to (Figure 3a). This analysis found no main effect of adaptation condition (*F* = 1.95 *p* > .1) or baseline (*F* < 1), but, as expected, found a significant main effect of head orientation (*F*(4, 56) = 217.29, *MSE* = 332.84, *p* < .001). There was no condition x baseline interaction (*F* = 1.86, *p* > .1), condition x orientation interaction (*F* = 1.32, *p* > .2), baseline x orientation interaction (*F* = 1.56, *p* > .2), or three-way interaction between these factors (*F* < 1). Consequently, the remaining analyses compare the effects of each adaptation condition (bodies, heads, and chairs) against the average of their individual corresponding baselines (2 and 4).

Following adaptation to left and right oriented bodies (same-social adaptor), but not adaptation to left and right oriented heads (different-social adaptor) or chairs (non-social adaptor), participants showed an increased tendency to categorize 6º left and 6º right oriented probe bodies as “direct” (Figure 3b). A 3x2x5 repeated measures ANOVA with factors of adaptor type, adaptation, and orientation showed a significant main effect of adaptation (*F*(1, 14) = 25.74, *MSE* = 84.78, *p *< .001) and orientation (*F*(4, 56) = 277.39, *MSE* = 276.09, *p *< .001), but no effect of adaptor type (*F *= 1.56, *p* > .2). Crucially, however, this analysis showed a significant adaptor type x adaptation interaction (*F*(1.36, 19) = 4.14, *MSE* = 187.35, *p *< .05) suggesting that the overall effects of adaptation are significantly different for the three adaptor types. There was also a borderline significant adaptor type x orientation interaction (*F*(8, 122) = 1.95, *MSE* = 77.21, *p *= .059) and a significant adaptation x orientation interaction (*F*(2.92, 40.87) = 4.31, *MSE* = 119.98, *p* < .01). Importantly, there was a strong trend toward a significant three-way interaction between adaptor type, adaptation, and orientation (*F*(8, 112) = 1.91, *MSE* = 70.34, *p *= .06). This suggests that the effects of adaptation are different for the three adaptor types.

To investigate these interactions further, individual 2x5 ANOVAs investigating adaptation and body orientation were conducted for each of the adaptation conditions. Consistent with previous research (Lawson et al., 2009), body adaptors produced a significant main effect of adaptation (*F*(1, 14) = 12.25, *MSE* = 275.99, *p* < .005), whereas heads (*F* = 3.53, *p* > .08) and chairs (*F* < 1) did not. All three adaptor types produced a significant main effect of orientation (Bodies *F*(2.46, 34.38) = 168.07, *MSE* = 138.13, *p* < .001; Heads *F*(2.57, 35.93) = 149.69, *MSE* = 289.70, *p* < .001, and Chairs *F*(4, 56) = 242.70, *MSE* = 106.50, *p* < .001). Furthermore, body adaptors produced a significant adaptation x orientation interaction (*F*(4, 56) = 4.52, *MSE* = 82.36, *p *< .005), whereas heads and chairs did not (*F*s < 1). Paired *t*-tests investigating the interaction between adaptation and body direction for the body adaptor condition showed that, consistent with the predictions of this paper, adaptation to an alternating sequence of 20º left and 20º right oriented bodies produced an *increased* tendency to call 6º left and 6º right probe bodies “direct” (L6° *t*(14) = 1.10, *p* < .001; R6° *t*(14) = 0.94, *p* = .036), although the latter narrowly misses Bonferroni correction. There was also a corresponding increase in “direct” responses to direct (0º) probes (Direct *t*(14) = 3.16, *p* < .005), and no significant effect for left 12º (*t*(14) = 1.31, *p* > .2), or right 12º probes (*t* < 1).

Thus far, it has been shown that adaptation transfers between adaptors of the same social cue type (i.e., bodies adapting bodies) but not between different-social adaptors (heads to bodies) or non-social adaptors (chairs to heads). These results are consistent with the hypothesis that body direction aftereffects arise from cells tuned to bodies alone. Finally, to ensure that the *effects of adaptation* produced by the body adaptors is significantly different from the head adaptors and the chair adaptors, three additional repeated measures ANOVAs were conducted to compare the effects of adaptation to chairs and bodies, heads and bodies, and finally, heads and chairs. The results can be seen in Table 1 and confirm that only bodies compared to either of the other adaptation conditions produces a significant three-way adaptor type x adaptation x orientation interaction. When comparing the effects of adapting to heads with adapting to chairs there is no adaptor type x adaptation interaction or three-way adaptor type x adaptation x orientation interaction, thus the effects of non-social (chairs) and different-social (heads) adaptors are indistinguishable.

## DISCUSSION

In Experiment 1 participants showed an increased tendency to categorize small angles of left and right oriented heads as “direct” following adaptation to 20º left and 20º right oriented heads (same-social adaptor), whereas after adapting to 20º left and 20º right oriented bodies (different-social adaptor) or 20º left and 20º right oriented chairs (non-social adaptor), they did not. Similarly, Experiment 2 demonstrated an increased tendency to categorize small angles of left and right oriented bodies as “direct” following adaptation to alternating 20º left and 20º right oriented bodies (same-social adaptor) but not after adapting to 20º left and 20º right oriented heads (different-social adaptor) or chairs (non-social adaptor). Additional analyses show that in both Experiment 1 and Experiment 2 the effects of adaptation are driven by the same-social adaptors whereas the different-social and non-social adaptation conditions do not differ from one another. Therefore, these results suggest that the adaptation aftereffects observed here arise from direction-selective cells tuned to heads and bodies alone.

The aftereffects reported in the same-social adaptor condition for both experiments generalize across a 25% size change between adaptor and probe images, meaning that these effects are unlikely to reflect adaptation of low-level image properties. Furthermore, while the previous head and body adaptation experiments (Lawson et al., 2009, 2011) demonstrate adaptation transfers across a change in identity (and gender) between the top-up adaptor and probe image, the 10 adaptor identities used were the same 10 identities that featured as the probe images. The results reported here go further in demonstrating identity-invariant yet direction-specific aftereffects, since the same-social adaptor condition in both experiments consist of six completely novel identities to those used as probes. Taken together, our results likely reflect adaptation of “high-level” representations of head and body direction localized to the ventral visual stream (Calder et al., 2007; Carlin, Calder, Kriegeskorte, Nili, & Rowe, 2011; Taylor et al., 2010). While the primary focus of this work was the adaptation of cues to social attention, it is interesting to speculate whether chair direction adaptors would have produced similar category-specific aftereffects for categorization of chair probes. Indeed, it’s unclear at present whether the category-specific direction aftereffects found here imply dedicated social-orienting mechanisms, or simply view-dependent representations of all objects. Future studies should explore adaptation to orientation within and across different object categories to garner new insight into the view-invariance/dependence of high-level object categories.

It has recently been argued that some high-level aftereffects, especially those that seem to transfer between face and non-face categories (Dennett, Edwards, & McKone, 2012; Ghuman, McDaniel, & Martin, 2010; Hills, Elward, & Lewis, 2010; Javadi & Wee, 2012), may actually represent biases such as perceptual contrast effects or a shift in the participant’s decision criterion (Storrs, 2015; Storrs & Arnold, 2012). If it were the case that adapting to the abstract concept of “leftness” or “rightness” was able to shift a participant’s decision criterion for reporting the direction of all probe stimuli, we would have expected the non-social chair stimuli in both experiments to produce aftereffects in the predicted direction and of the same magnitude as the same-social adaptor condition, which was not the case. Therefore, the specificity of these aftereffects suggests they reflect genuine changes to perceptual representations. Nonetheless, it’s interesting to consider why representations of direction seem strongly category-specific, whereas gender and identity and configural representations seem less so. Eye gaze, head direction, and body orientation aftereffects are all best accounted for by a multichannel coding system (Calder et al., 2008; Lawson et al., 2009, 2011), with the adaptor most likely engaging the singe pool of cells most selective for that particular direction along the single dimension of orientation. In contrast, gender, identity, and configural attributes of social stimuli are, to some extent, opponent-coded (Leopold, O’Toole, Vetter, & Blanz, 2001; Pond et al., 2013; Rhodes, Jeffery, Watson, Clifford, & Nakayama, 2003; though see Storrs & Arnold, 2012), which may suggest broad and distributed co-activation of many pools of cells collectively representing the adaptor “extreme” across multiple dimensions. This distinction in the way in which directional and non-directional aspects of social stimuli are represented likely contributes to the differences in category-specificity of these cues, and their susceptibility to non-perceptual biases. Further studies will be necessary to explore the underlying mechanisms of adaptation to different attributes of social cues.

We note that in Experiment 1 the non-social adaptor condition (chairs) produced a significant interaction between adaptation and orientation and follow-up analyses revealed that this was due to a small but significant increase in “direct” responses to direct oriented heads and a *decrease* in “direct” responses to right 6º oriented heads. While surprising, these effects are unlikely to reflect adaptation per se. The predicted aftereffects following alternating adaptation of channels (pools of cells) representing left and right oriented stimuli would comprise an increased tendency to categorize left and right oriented probes as “direct” (Calder et al., 2008; Lawson et al., 2009, 2011). Inconsistent with the predicted effects of adaptation, the chair adaptor condition in Experiment 1 actually produced a decrease in “direct” responses to 4º right head probes whereas the predicted aftereffects *were* found for the same-social adaptor condition in both experiments 1 and 2. Additionally, the effect of adaptation to chairs is statistically indistinguishable from the different-social adaptor condition (bodies), which shows no effects of adaptation. Therefore, while it is acknowledged that effect of chairs in Experiment 1 is somewhat anomalous, it is not thought to be an effect of adaptation or to undermine the reported effects of adaptation in the same-social condition.

Importantly, our results suggest that head and body direction aftereffects in humans do not arise from cells analogous to the “social attention” cells discovered in the macaque STS (Perrett et al., 1992). That is, they do not reflect the engagement (and hence adaptation) of direction-selective cells that respond to *both* head and body cues. That said, these experiments are silent with regards to the existence of social attention cells in humans, they can only speak to the types of neural representations which give rise to head and body orientation aftereffects. Perrett and colleagues’ (1992) model of cue integration in macaques predicts that where cues from the head and body are both visible and oriented in opposite directions, responses from head inputs inhibit the responses of the body inputs. This hierarchical response modulation of social attention cells is most relevant in situations where information from two cues are in conflict with one another and evidence from single-cell recording suggests that when directionally consistent head and body cues are presented in isolation, they engage the same social attention cell by equivalent amounts (Perrett et al., 1992). Nonetheless, it is inherent in this model that cues from the head are more important in signaling direction of attention than cues from the body (and cues from the eyes, which inhibit inputs from head cells, are most meaningful of all). However, the lack of adaptation in the different-social adaptor condition of Experiment 2 not only confirms the results of Experiment 1 (that these aftereffects arise from cells responsive to heads and bodies alone), but also suggests that putative social attention cells do not seem to be any more effectively engaged by head stimuli than body stimuli. This is consistent with studies that, in the context of gaze and head direction cues, suggest that information from both cues exhibit equal influence in determining the locus of others’ attention (Langton, Watt, & Bruce, 2000).

In conclusion, we have shown that visual representations of where another person is attending (signaled by the head and the body) are coded by high-level direction-specific representation of head and body *alone*. Our results suggest that these aftereffects do not arise from “social attention cells” or abstract representations of “left” and “right.”

## Notes

No potential conflict of interest was reported by the authors.
